# The association and a potential pathway between gender-based violence and induced abortion in Thai Nguyen province, Vietnam

**DOI:** 10.3402/gha.v5i0.19006

**Published:** 2012-11-29

**Authors:** Phuong Hong Nguyen, Son Van Nguyen, Manh Quang Nguyen, Nam Truong Nguyen, Sarah Colleen Keithly, Lan Tran Mai, Loan Thi Thu Luong, Hoa Quynh Pham

**Affiliations:** 1 Thai Nguyen University of Medicine and Pharmacy, Thai Nguyen, Vietnam; 2 International Food Policy Research Institute, Hanoi, Vietnam; 3 Institute of Social and Medical Studies, Hanoi, Vietnam

**Keywords:** Gender-based violence, abortion, contraceptive use, unintended pregnancy, Thai Nguyen, Vietnam

## Abstract

**Background:**

Gender-based violence (GBV) has profound adverse consequences on women's physical, mental, and reproductive health. Although Vietnam has high rates of induced abortion and GBV, literature examining this relationship is lacking.

**Objective:**

This study examines the association of GBV with induced abortion among married or partnered women of reproductive age in Thai Nguyen province, Vietnam. In addition, we explore contraceptive use and unintended pregnancy as mediators in the pathway between GBV and induced abortion.

**Design and methods:**

Data were drawn from a cross-sectional survey of 1,281 women aged 18–49 years in four districts of Thai Nguyen province. Bivariate and multivariate logistic regression analyses were applied to examine the associations between lifetime history of GBV, contraceptive use, unintended pregnancy, induced abortion, and repeat abortion, controlling for other covariates.

**Results:**

One-third of respondents had undergone induced abortion in their lifetime (33.4%), and 11.5% reported having repeat abortions. The prevalence of any type of GBV was 29.1% (17.0% physical violence, 10.4% sexual violence, and 20.1% emotional violence). History of GBV was associated with induced abortion (OR=1.61, 95% CI: 1.20–2.16) and repeat abortion (OR=2.22, 95% CI: 1.48–3.32). Physical violence was significantly associated with induced abortion, and all three types of violence were associated with repeat abortion. Abused women were more likely than non-abused women to report using contraceptives and having an unintended pregnancy, and these factors were in turn associated with increased risk of induced abortion.

**Conclusions:**

GBV is pervasive in Thai Nguyen province and is linked to increased risks of induced abortion and repeat abortion. The findings suggest that a pathway underlying this relationship is increased risk of unintended pregnancy due in part to ineffective use of contraceptives. These findings emphasize the importance of screening and identification of GBV and incorporating women's empowerment in reproductive health and family planning programs.

Violence against women, also known as gender-based violence (GBV), is “now widely recognized as a serious human rights abuse” as well as “an important public health problem that concerns all sectors of society” (1). Internationally, attempts have been made to redress this issue, with the United Nations Declaration of Elimination of Violence against Women in 1993. In 2005, the World Health Organization (WHO) conducted a multi-country study on violence against women, which highlighted that GBV prevalence is far greater than that indicated by research prior to 1999 (1). Factors contributing to the hidden nature of GBV include victims’ fear of retaliation or other consequences, gender norms that reinforce women's subordination to their male counterparts, and social norms that justify GBV as ‘normal’ (2). The adverse health consequences of GBV are not limited to the physical and mental wellbeing of a woman but also encompass her reproductive health (1, 3). Domestic violence against women in their childbearing years can lead to a range of health problems for both mothers and their babies, including late prenatal care during pregnancy, adverse pregnancy outcomes (4), somatic disorders, neonatal problems, infant mortality (5), and even maternal death (6). Specifically, literature has shown the negative effects of domestic violence against women on sexual autonomy, unintended pregnancy (3, 7), spontaneous abortion, and induced abortion (8, 9).

Previous studies show that GBV remains a widespread problem in Vietnam with indications that it may be on the increase (10). According to a 2010 nationally representative study on domestic violence, 58% of Vietnamese women have experienced some form of violence in their lifetime, with prevalence rates of physical, sexual, and emotional violence at 32, 10, and 54%, respectively (11).

In addition to GBV, maternal health in Vietnam suffers from conspicuously high abortion rates (an average of 2.5 induced abortions in a woman's reproductive life), which persist despite the country's high contraceptive prevalence rate (78% among married couples) (12, 13). While no official data on unsafe abortion are available for Vietnam, a 2000–2001 study found that unsafe abortion was the direct cause of 11.5% of maternal deaths in seven Vietnamese provinces (14).

Due to the scope of violence against women and its importance for reproductive health, research has increasingly focused on examining the association between GBV and abortion. Studies in developing countries, such as India (15), Tanzania (16), Bangladesh (9), China (17), and Uganda (7), have identified GBV as a significant risk factor for induced abortion. However, the literature examining the determinants of induced abortion in Vietnam is yet to explore GBV as a possible predictor (18, 19). In light of this gap, this study aims to document the prevalence of GBV among married women of reproductive age in Thai Nguyen province, Vietnam, and to examine the association of GBV with induced abortion and repeat induced abortion.

The study also aims to explore an underlying pathway linking GBV and induced abortion in which contraceptive use and unintended pregnancy act as mediating factors. Research suggests that by creating an environment of fear and intimidation, increasing psychological stress, and reducing access to services and support, GBV may limit women's control over their fertility, thereby reducing contraceptive use or interrupting the effective use of contraceptives (20–22). It has also been posited that victims of GBV may feel unprepared or have reduced desire to raise a child in an abusive environment, which could increase the likelihood that pregnancies are unintended and terminated (22, 23). Further, women may be forced into having an abortion by their abusive partners (16, 22, 24). We hypothesized that GBV reduces women's contraceptive use, thereby increasing their risk of unintended pregnancy and, consequently, induced abortion (Fig. 1).

**Fig. 1:**
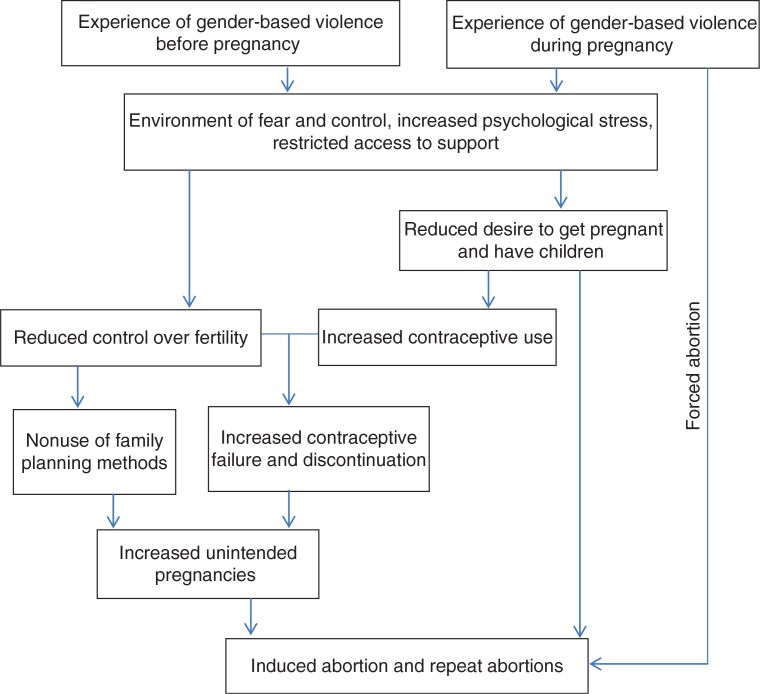
Conceptual model of a potential pathway between gender-based violence (GBV) and induced abortion.

**Implications for policy and practice**

- Assessment and treatment of GBV should be integrated into standard reproductive health and family planning services in Vietnam. To do so successfully, health providers need guidelines and training in screening, documenting, and treating GBV.
- Investment is needed to establish shelters and support centers for victims of GBV that provide both protection and counseling services. Linkages should be developed between these centers and reproductive health and family planning services.
- Programs are needed to address gender inequality by advancing women's empowerment, socioeconomic status, and education as a means for curbing GBV.
- It is necessary to increase public awareness of the physical, reproductive, mental, and societal consequences of GBV and to shift norms of acceptance of GBV.
- The capacity of the police and local authorities to uphold GBV policies and legislation should be strengthened.

## Methods

### Design and settings

Data were drawn from a cross-sectional household survey conducted as part of a project entitled ‘Primary Health Improvement Initiative in Thai Nguyen Province, Vietnam.’ By providing evidence for health policy planning, the project aimed to advance the national effort to attain universal access to family planning and safe abortion services in Vietnam. The survey was conducted between February and May 2011 in four of the nine districts of Thai Nguyen, a province in northern Vietnam with high proportions of indigent and ethnic minority women. These districts account for 52% of the province's total population and are representative of both urban (Thai Nguyen city) and rural areas (Dai Tu, Dinh Hoa, and Vo Nhai districts).

### Sampling and data collection

A representative sample of married or partnered women aged 18–49 years was selected from the four districts by a two-stage cluster sampling technique. The first stage involved selection of 40 communes as primary sampling units, with probability of inclusion proportional to the size of the population in each district. In the second stage, 40 individual respondents were selected in each commune via systematic random sampling.

Data were collected using face-to-face interviews using a structured questionnaire. The survey collected information from respondents on sociodemographic background, sexuality and obstetric history, knowledge, attitudes and practices related to contraception, fertility preferences, history of GBV, attitudes towards and access to family planning and reproductive health services, history of abortion, and experience with abortion services. Interviewers were trained medical doctors with sufficient technical ability to distinguish among induced abortions, stillbirths, and miscarriages. A total of 1,540 women completed the survey. Among those, 1,281 women were married or living with partner and were included in this analysis.

### Variables of interest

The outcomes were self-reported lifetime history of induced abortion and repeat (two or more) induced abortion. The main independent variables were self-reported lifetime experience of any GBV as well as physical violence, sexual violence, and emotional violence, based on the WHO definition (1) and adapted for the Vietnam context (11). Physical violence was assessed in terms of 11 items: slapping, throwing things, pushing or shoving, hair pulling, hitting, kicking, dragging, beating, choking, burning, and threatening with or using a weapon such as a knife or scissors. Sexual violence was assessed in terms of three items: having sexual intercourse against the respondent's will, partner using excessive physical force during sexual intercourse, and being forced to engage in sexually degrading acts. Emotional violence was assessed in terms of three items: insults or degrading activities, belittlement or humiliation, and scaring the respondent (including threats of violence). Lifetime occurrence of any kind of violence was defined as experience of any act of violence up to the date of the interview, perpetrated by a current or former husband/partner. In addition, because women reporting a particular type of violence (e.g. physical) could also be exposed to another form of violence (e.g. sexual), lifetime history of each type of violence was assessed independently.

Potential mediating explanatory factors included contraceptive use and unintended pregnancy. To measure contraceptive use, respondents were asked whether they had ever used any contraceptive method in their lifetime, and, if so, the type(s) of method used. Responses were categorized into three groups: use of any contraceptives (yes/no), use of female-only methods (yes/no), and use of couple methods (yes/no). Female-only methods included oral pills, intrauterine devices (IUDs), injectables, implants, and female sterilization. Couple methods included those that require at least the awareness and a certain degree of support and cooperation from husbands, such as male and female condoms, diaphragms, withdrawal, breastfeeding, and periodic abstinence. Unintended pregnancy was assessed by asking respondents if they would describe their current pregnancy or any pregnancy in the past as unintended (yes/no).

Additional explanatory variables included as control variables in the multivariate analyses were women's age at interview (18–24 years/25–34 years/35–49 years); ethnicity (Kinh majority/other ethnic minority groups); highest level of education completed [primary school (grades 1–5)/secondary school (grades 6–9)/high school (grades 10–12)/college or higher]; main occupation (farmers/other occupations); area of residence (rural/urban); household social economic status (SES) (lowest/lower/middle/higher/highest); number of living children (≤1 child/2 children/≥3 children); and age at first intercourse (14–19 years/20–24 years/≥25 years). The household SES index was constructed using principal components analysis using asset data such as house and land ownership, housing quality, access to services (water, electricity, gas, and sanitation services), and household assets (different types of durable goods, productive assets, animals and livestock) (25, 26). Household SES categories were formed by dividing the index into quintiles. Due to its high correlation with occupation, area of residence was not included in the multivariate analyses.

### Statistical analyses

Data were analyzed using Stata 11 (27). Descriptive statistics were used to report the study population's background characteristics and to estimate prevalence of GBV and induced abortion. Proportions and chi-squared test were used to examine the binary associations between GBV and contraceptive use, unintended pregnancy, and induced abortion outcomes. Multivariate logistic regression analysis was used to explore the association of GBV variables with lifetime history of induced abortion and repeat induced abortion, controlling for other covariates. In addition, multivariate logistic regression analyses were run to study the relationship between GBV and contraceptive use and unintended pregnancy. Odds ratios (OR) with 95% confidence intervals (95% CI) are presented for the final models.

### Ethical considerations

Ethical approval for this study was obtained from the Institutional Review Board of the Institute of Social and Medicine Studies in Vietnam. Written informed consent was obtained from all respondents, and measures were taken to ensure participants’ confidentiality and privacy throughout the study process.

## Results

The overall response rate was high at 95%. The internal non-response rates of the study's sensitive questions – including those related to contraceptive use, unintended pregnancy and induced abortion, and exposure to GBV – were very low, ranging from 0.3–2.1%. The limited number of respondents with missing data on these variables was unlikely to have greatly affected the results.

Table 1 provides an overview of the prevalence of and associations between lifetime history of GBV with contraceptive use, unintended pregnancy, induced abortion, and repeat induced abortion. One-third of women reported undergoing at least one induced abortion, and 11.5% reported having two or more induced abortions in their lifetime. The prevalence of GBV was relatively high, with 29.1% of women reporting experiencing any types of violence by their husband/partner, 17.0% reporting physical violence, 10.4% reporting sexual violence, and 20.1% reporting emotional violence. About two-thirds of respondents had used contraception, with more women reporting use of female-only methods (48.8%) than couple methods (19.9%). Close to one-quarter of women (23.6%) reported having an unintended pregnancy currently or in the past.

**Table 1:** Prevalence of lifetime history of gender-based violence (GBV) and association between GBV and lifetime contraceptive use, unintended pregnancy, and induced abortion

		Lifetime history of contraceptive use and unintended pregnancy (*n*=1,260)				Lifetime history of induced abortion (*n*=1,254)	
			
	Prevalence (*n*=1,281)	Contraceptive use (*n*=865, 68.66%)	Use of female contraceptive method (*n*=615, 48.77%)	Use of couple contraceptive method (*n*=251, 19.89%)	Unintended pregnancy (*n*=297, 23.57%)	Abortion (*n*=421, 33.57%)	Repeat abortion (*n*=144, 11.48%)
	
Lifetime history of GBV	Percent (95% CI)	Percent	Percent	Percent	Percent	Percent	Percent
Any GBV							
No	70.95 (68.44–73.46)	65.70	44.39	20.98	21.05	30.54	9.07
Yes	29.05 (26.54–31.56)	77.57***	50.00***	17.14	29.56**	40.93***	17.31***
Physical GBV							
No	83.02 (80.94–85.09)	67.32	46.24	20.77	21.88	31.41	9.69
Yes	16.98 (14.91–19.06)	78.02**	62.15***	15.25+	31.60**	44.13***	20.19***
Sexual GBV							
No	89.62 (87.93–91.30)	68.25	47.89	19.92	22.51	32.73	10.40
Yes	10.38 (8.69–12.07)	76.07+	56.41+	19.66	32.82**	40.46+	20.61**
Emotional GBV							
No	79.87 (77.66–82.09)	66.99	45.72	21.02	22.20	32.22	9.56
Yes	20.13 (17.91–22.34)	77.73**	61.97***	15.02*	29.08*	38.74*	18.97***

+ *p*<0.10

* *p*<0.05

** *p*<0.01

*** *p*<0.001.

Lifetime history of induced abortion and repeat induced abortion were significantly associated with having experienced GBV (Table 1). Women who had undergone an abortion were significantly more likely to report experiencing any types of violence, physical violence, and emotional violence and were marginally more likely to experience sexual violence. Those reporting repeat abortion had a greater likelihood of experiencing any types of violence as well as all forms of GBV. Table 2 summarizes the demographic characteristics of the study sample as well as the association between these characteristics with induced abortion and repeat abortion. As expected, women who were older and had more children were more likely to have experienced abortion. Women who worked as farmers were less likely to report abortion compared to women having other jobs (30.1% vs. 39.9% for induced abortion and 10.1% vs. 14.1% for repeat abortion). Women who had first intercourse before age 25 were more likely to experience abortion. In addition, women reporting use of contraceptives also had increased likelihood of having experienced abortion. Women with higher SES status were also more likely to report experiencing any abortion and repeat abortion.

**Table 2:** Demographic characteristics, contraceptive use, unintended pregnancy, and lifetime history of induced abortion among study participants

		Lifetime history of abortion (*n*=1,254)	
		
	Prevalence (*n*=1,281)	Abortion (*n*=421, 33.57%)	Repeat abortion (*n*=144, 11.48%)
	
Explanatory variables	Percent (95% CI)	Percent	Percent
Age of respondent (years)			
18–24	9.40 (7.79–11.00)	10.09	0.00
25–34	34.38 (31.77–36.70)	23.00	5.16
35–49	56.22 (53.50–58.95)	43.50***	16.92***
Ethnicity			
Kinh	67.37 (64.80–69.94)	34.75	13.00
Other (ethnic minority)	32.63 (30.06–35.20)	31.13	8.33*
Education			
Primary school	10.62 (8.93–12.31)	28.68	8.82
Secondary school	50.27 (47.53–53.01)	34.34	11.39
High school	23.50 (21.17–25.82)	30.17	11.53
College or higher	15.61 (13.62–17.60)	39.79+	13.61
Occupation			
Farmer	64.77 (62.15–67.37)	30.14***	10.09*
Other	35.23 (32.61–37.85)	39.91	14.06
Area of residence			
Rural	64.64 (62.02–67.26)	29.88***	8.52***
Urban	35.36 (32.74–37. 99)	40.32	16.89
Household SES			
Lowest	20.02 (17.83–22.23)	25.70	7.23
Low	20.03 (17.83–22.23)	28.97	7.94
Middle	19.95 (17.76–22.15)	33.47	10.76
Higher	20.03 (17.83–22.23)	37.55	13.83
Highest	19.95 (17.76–22.15)	42.28***	17.89***
Number of living children (range: 0–5)			
≤1	34.35 (31.74–36.95)	20.58	4.60
2	52.30 (49.56–55.04)	38.36	14.48
≥3	13.35 (11.48–15.21)	46.20***	16.37***
Age at first intercourse (years)			
14–19	27.97 (25.50–30.43)	34.76	11.11
20–24	52.63 (49.89–55.38)	35.26	12.61
25–45	19.40 (17.23–21.58)	26.36*	8.79
Ever used contraception			
No	31.34 (28.64–34.04)	8.20	1.64
Yes	68.66 (65.96–71.36)	35.49***	12.08*
Ever used female contraceptive methods			
No	51.23 (48.32–54.14)	23.17	5.88
Yes	48.77 (45.89–51.68)	44.85***	17.18***
Ever used couple contraceptive methods			
No	80.11 (77.78–82.43)	34.64	12.78
Yes	19.89 (17.57–22.22)	31.08	6.31**
Ever had unintended pregnancy			
No	76.43 (74.07–78.80)	32.69	11.32
Yes	23.57 (21.20–25.93)	50.82**	14.75

+ *p*<0.10

* *p*<0.05

** *p*<0.01

*** *p*<0.001.

The multivariate logistic regression models of the relationship between GBV and abortion outcomes are presented in Tables 3 and 4. After adjusting for potential confounders, women who experienced any form of GBV were 1.61 times (95% CI: 1.20–2.16) more likely to report having an induced abortion and 2.22 times (95% CI: 1.48–3.32) more likely to report having repeat induced abortion. When considering the odds of induced abortion by the three subtypes of GBV, the adjusted OR for physical violence was 1.91 (95% CI: 1.34–2.72), while the associations with the two other subtypes (sexual and emotional violence) were not statistically significant. All three subtypes of GBV were positively associated with repeat abortion, with adjusted OR ranging from 2.28 to 2.50 (Table 4).

**Table 3:** Multivariate logistic regression models for the relationship between any kind of gender-based violence (GBV) and induced abortion

	Ever had abortion (*n*=1,118)	Ever had repeat abortion (*n*=1,118)
	
Explanatory variables	OR (95% CI)	OR (95% CI)
Ever experienced any GBV		
No^r^	1.00	1.00
Yes	1.61** (1.20–2.16)	2.22*** (1.48–3.32)
Ever used contraception		
No^r^	1.00	1.00
Yes	4.10** (1.56–10.77)	4.81 (0.64–36.22)
Ever had unintended pregnancy		
No^r^	1.00	1.00
Yes	2.69** (1.44–5.01)	1.82 (0.74–4.43)
Age of respondent (years)		
18–24^r^	1.00	1.00
25–34	3.07* (1.31–7.20)	
35–49	7.49*** (3.11–18.06)	3.39*** (1.91–6.02)
Ethnicity		
Kinh	0.76+ (0.56–1.04)	1.01 (0.62–1.64)
Other^r^	1.00	1.00
Education		
Primary school^r^	1.00	1.00
Secondary school	1.17 (0.72–1.89)	1.16 (0.53–2.53)
High school	0.83 (0.46–1.50)	0.95 (0.38–2.38)
College or higher	1.19 (0.60–2.34)	1.12 (0.40–3.11)
Occupation		
Farmer	0.61* (0.39–0.93)	0.95 (0.52–1.74)
Other^r^	1.00	1.00
Household SES		
Lowest^r^	1.00	1.00
Lower	1.01 (0.64–1.61)	0.87 (0.40–1.87)
Middle	1.42 (0.88–2.28)	1.45 (0.69–3.04)
Higher	1.46 (0.87–2.45)	1.94+ (0.88–4.23)
Highest	1.55 (0.86–2.81)	2.77* (1.17–6.58)
Number of living children		
≤1^r^	1.00	1.00
2	1.39 (0.94–2.05)	1.83+ (0.95–3.53)
≥3	1.90* (1.11–3.25)	2.19+ (0.96–5.00)
Age at first sexual intercourse (years)		
14–19	1.63* (1.03–2.58)	1.27 (0.65–2.50)
20–24	1.61* (1.10–2.36)	1.37 (0.78–2.43)
25–45^r^	1.00	1.00

+ *p*<0.10

* *p*<0.05

** *p*<0.01

*** *p*<0.001.

r Reference group.

**Table 4:** Multivariate logistic regression models for the relationship between physical, sexual, and emotional gender-based violence (GBV) and induced abortion

	Model 1^1^,^2^		Model 2		Model 3	
			
	Ever had abortion (*n*=1,118)	Ever had repeat abortion (*n*=1,118)	Ever had abortion (*n*=1,118)	Ever had repeat abortion (*n*=1,118)	Ever had abortion (*n*=1,118)	Ever had repeat abortion (*n*=1,118)
	
Explanatory variables	OR (95% CI)	OR (95% CI)	OR (95% CI)	OR (95% CI)	OR (95% CI)	OR (95% CI)
Ever experienced GBV						
Physical GBV						
No^r^	1.00	1.00				
Yes	1.91*** (1.34–2.72)	2.50*** (1.58–3.96)				
Sexual GBV						
No^r^			1.00	1.00		
Yes			1.35 (0.88–2.06)	2.28** (1.34–3.88)		
Emotional GBV						
No^r^					1.00	1.00
Yes					1.31 (0.94–1.83)	2.36*** (1.53–3.63)
Ever used contraception						
No^r^	1.00	1.00	1.00	1.00	1.00	1.00
Yes	4.31** (1.64–11.3)	5.38 (0.71–40.6)	4.28** (1.63–11.2)	5.12 (0.68–38.5)	4.28** (1.63–11.2)	5.17 (0.69–39.0)
Ever had unintended pregnancy						
No^r^	1.00	1.00	1.00	1.00	1.00	1.00
Yes	2.80** (1.50–5.24)	1.87 (0.76–4.61)	2.72** (1.46–5.07)	1.84 (0.76–4.47)	2.74** (1.47–5.12)	1.78 (0.72–4.37)

+ *p*<0.10

* *p*<0.05

** *p*<0.01

*** *p*<0.001.

1 The main independent variables for models 1, 2, and 3 were physical GBV, sexual GBV, and emotional GBV, respectively. These independent variables were highly correlated and were therefore examined in separate models.

2 Models control for the following (not shown): age, ethnicity, education, occupation, household SES, number of living children, and age at first intercourse.

r Reference group.

To assess contraceptive use and unintended pregnancy as potential mediators on the pathway between GBV and induced abortion, we examined the relationship between these variables and the independent variables of interest (GBV variables) and the dependent variables of interest (induced abortion outcomes). Findings show that both contraceptive use and unintended pregnancy were associated with GBV in both bivariate analyses (Table 1) and multivariate analyses (Table 5). Controlling for potential confounders, women who were exposed to any form of violence were 1.82 times (95% CI: 1.30–2.47) more likely to report contraceptive use and 1.66 times (95% CI: 1.11–2.11) more likely to report unintended pregnancy than unexposed women. Abused women were 1.76 times (95% CI: 1.33–2.33) more likely to report using female-only contraceptive methods than those unexposed to violence. In contrast, the association between GBV and use of couple contraceptive methods was statistically insignificant. In addition to GBV, the reproductive health mediators were also associated with induced abortion outcomes. Use of any form of contraception and use of female-only methods were associated with increased likelihood of having an induced abortion and repeat abortion (Tables 2 and 3). In addition, abortion-seekers were more likely to report having an unintended pregnancy in their lifetime. Multivariate logistic regression analyses show that these relationships remained even after adjusting for potential confounders (Table 3).

**Table 5:** Multivariate logistic regression models for the relationship between any kind of gender-based violence (GBV) and contraceptive use and unintended pregnancy

	Contraceptive use			
		
	Ever used contraception (*n*=1,138)	Ever used female only contraceptive method (*n*=1,124)	Ever used couple contraceptive method (*n*=1,124)	Ever had unintended pregnancy (*n*=1,211)
	
Explanatory variables	OR (95% CI)	OR (95% CI)	OR (95% CI)	OR (95% CI)
Ever experienced any GBV				
No^r^	1.00	1.00	1.00	1.00
Yes	1.82*** (1.33–2.49)	1.76*** (1.33–2.33)	0.89 (0.62–1.27)	1.66** (1.22–2.26)
Age of respondent (years)				
18–24^r^	1.00	1.00	1.00	1.00
25–34	1.70* (1.05–2.76)	2.35** (1.39–3.96)	0.65 (0.37–1.15)	0.71 (0.36–1.43)
35–49	1.44 (0.84–2.48)	2.66*** (1.51–4.68)	0.43** (0.22–0.81)	0.41* (0.20–0.87)
Ethnicity				
Kinh	1.09 (0.82–1.46)	0.92 (0.69–1.22)	1.36+ (0.95–1.96)	1.01 (0.72–1.41)
Other^r^	1.00	1.00	1.00	1.00
Education				
Primary school^r^	1.00	1.00	1.00	1.00
Secondary school	0.97 (0.62–1.54)	0.89 (0.58–1.37)	1.13 (0.61–2.09)	1.16 (0.73–1.86)
High school	0.78 (0.45–1.33)	0.85 (0.51–1.43)	0.90 (0.44–1.81)	0.76 (0.42–1.38)
College or higher	1.00 (0.52–1.93)	0.60 (0.36–1.12)	1.76 (0.81–3.84)	0.82 (0.40–1.68)
Occupation				
Farmer	0.98 (0.64–1.50)	0.93 (0.62–1.38)	1.07 (0.66–1.73)	1.00 (0.62–1.60)
Other^r^	1.00	1.00	1.00	1.00
Household SES				
Lowest^r^	1.00	1.00	1.00	1.00
Lower	1.30 (0.86–1.96)	1.14 (0.76–1.70)	1.27 (0.72–2.23)	0.88 (0.56–1.39)
Middle	1.80** (1.16–2.78)	1.47+ (0.97–2.24)	1.34 (0.76–2.37)	0.81 (0.50–1.32)
Higher	1.53+ (0.94–2.48)	0.91 (0.57–1.46)	2.21** (1.22–4.02)	1.57+ (0.93–2.64)
Highest	2.15** (1.21–3.81)	1.36 (0.79–2.34)	1.90+ (0.96–3.74)	1.33 (0.70–2.49)
Number of living children				
≤1^r^	1.00	1.00	1.00	1.00
2	1.68** (1.18–2.39)	1.61** (1.15–2.25)	1.00 (0.67–1.51)	4.07*** (2.53–6.55)
≥3	1.80* (1.06–3.07)	1.82* (1.10–2.99)	0.83 (0.42–1.66)	18.05*** (9.84–33.12)
Age at first sexual intercourse (years)				
14–19	1.37 (0.89–2.10)	2.06*** (1.36–3.11)	0.53* (0.32–0.88)	1.26 (0.76–2.09)
20–24	1.37+ (0.96–1.96)	1.85*** (1.31–2.61)	0.63* (0.43–0.94)	1.13 (0.73–1.78)
25–45^r^	1.00	1.00	1.00	1.00

+ *p*<0.10

* *p*<0.05

** *p*<0.01

*** *p*<0.001.

r Reference group.

## Discussion

In parallel with socioeconomic development, Vietnam has seen significant gains in gender equality and women's advancement in recent years. However, Vietnamese culture remains deeply rooted in traditional Confucian principles that serve to lower women's status in relation to men, such as the declaration that wives should be subordinate to their husbands (28). Such inequities between men and women are a root cause of GBV, which is known to be perpetuated by a culture of silence in Vietnam (11). Contributing to this silence are gender norms that place women in the position of maintaining family harmony and call for women to protect the reputation of their husbands and families. GBV is considered a family and private issue, not to be discussed openly. This silence reduces public awareness of the problem and prevents abused women from getting support and protection they need (11).

This study provides evidence that GBV remains a formidable problem in Thai Nguyen province with serious reproductive health consequences for the women who live there. The prevalence rates of GBV in the present study were lower than the results from the 2010 national study on domestic violence (11) (e.g. 31% vs. 58% for any form of GBV), most likely due to variations between the two study populations. Compared to the national study, our sample had larger proportions of rural residents and ethnic minorities, and these populations may be more likely to underreport GBV.

Our findings demonstrate that women who experience GBV are at an increased risk for induced abortion and repeat induced abortion. After adjusting for potential confounders, the risk for repeat abortion remained for all three subtypes for GBV, while the risk of having an induced abortion remained significant for physical violence but not for sexual violence and emotional violence. These findings corroborate some of the findings from literature studying GBV and abortion. Findings from a study in Kenya (8) show that induced abortion was associated with all three subtypes of GBV. However, in Cameroon (29), the risk for induced abortion increased with physical and sexual violence but was not associated with emotional violence.

Evidence suggests that contraceptive use and unintended pregnancy are factors mediating the relationship between GBV and abortion. We found that GBV was positively associated with contraceptive use and unintended pregnancy, and, in turn, these factors were positively associated with induced abortion. These findings are consistent with the literature demonstrating that abused women are more likely to use contraception (5, 23, 30) and to have unintended pregnancy (9, 31) than their non-abused counterparts.

This pathway may be explained by reluctance on behalf of the abused women to raise a child in a violent setting, which would lead them to take matters into their own hands using contraception to avoid pregnancy/childbirth. This theory helps explain why use of female-only contraceptive methods was associated with abortion but not use of couple methods. Couple methods intrinsically require couple communication and cooperation, while female-only methods can be used without the partner's involvement or even knowledge. However, it has also been theorized that this finding may be due to men discovering that their partners are using contraception and then reacting violently, which would also lead to a positive association between contraceptive use and GBV (20).

While it appears paradoxical that high contraceptive use goes together with high unintended pregnancy and high abortion rates among abused women, this finding may be related to the influence of GBV on the effective use of contraception. The environment of fear and male dominance evident in abusive relationships may reduce women's ability to use contraception consistently and effectively, thereby increasing their risk of unintended pregnancy and abortion (22). In addition, evidence suggests that contraceptive failure is relatively common in Vietnam, where an estimated 36.7% of unintended pregnancies are the result of contraceptive failure, which helps explain the country's high rates of contraceptive use and abortion (32). Another recent publication from this study found that family planning services in Thai Nguyen remain limited in terms of counseling about method choice as well as contraceptive access and method availability (33). Inadequate family planning services may contribute to higher rates of contraceptive failure in this population.

The other logical hypothesis is that women exposed to GBV have reduced control over their reproductive choices due to reduced access to family planning or other fertility control resources and therefore experience higher rates of unintended pregnancy. Abortion may be one method by which these women regain control of their reproductive health. Finally, there remains the possibility that abusive partners have used violence or the threat of violence to coerce these women into having abortions.

This study contains limitations that should be taken into consideration. Due to the study's cross-sectional design, it is impossible to surmise causality from the findings. Only the associations between having experienced GBV and induced abortion can be inferred. Another limitation is the possibility of underreporting of history of GBV and induced abortion due to our reliance on retrospective reports as well as the sensitive nature of the questions. In particular, younger women are likely to have underreported their experience with abortion since having an abortion before the first birth is viewed as disgraceful in Vietnam. In addition, our data did not include single women, whose GBV experiences and induced abortion behaviors could be different from those of married women. However, the results can be generalized to married or partnered women of reproductive age in Thai Nguyen province as well as the wider region of northern Vietnam. Despite these limitations, the study findings have important implications for policy and practice in Vietnam.

The Vietnam family planning program has made impressive gains in increasing contraceptive use and reducing fertility (34). However, family planning may not be easy for women in violent relationships, and therefore policies and programs in Vietnam should consider the role of GBV in women's reproductive health. At present, there are no national treatment guidelines for health care providers to use in screening, treating, and referring GBV victims. Nationwide interventions should focus on providing guidelines and training to health workers for screening and treatment of GBV during routine family planning and antenatal care visits. Because abused women also need support and protection outside of health care settings, referrals should be provided to appropriate support centers if abuse is identified. As of now, these centers are severely lacking; it is critical to establish effective protection, support, counseling, and treatment services for abused women.

While the National Assembly issued the Law on Domestic Violence Prevention and Control in 2007, knowledge of the law is still lacking and implementation remains weak, especially in rural and poor areas (35). Furthermore, the culture of silence around GBV means that many of the serious consequences go unrecognized by policymakers and the community at large. Efforts should be made to increase public awareness of the physical, reproductive, mental, and societal consequences of GBV and to shift norms of acceptance of GBV. To transfer some of the burden away from women, it is important to hold police and local authorities responsible for the implementation of GBV policies and legislation.

## Conclusions

This study is among the first to provide evidence of a positive association between GBV and induced abortion in Vietnam. In addition, the study provides insight into the role that contraceptive use and unintended pregnancy play in the underlying pathway between GBV and induced abortion. Addressing Vietnam's high prevalence of GBV has the potential to significantly improve maternal, infant, and reproductive health outcomes. In particular, reducing GBV could lower the country's stubbornly high abortion rates. Our findings emphasize the importance of reducing gender inequality, integrating routine screening and treatment of GBV into reproductive health care, providing supportive services for victims of violence, and raising awareness of the extent and consequences of GBV. Additional research is needed to further the development and improvement of policies and programs. Qualitative studies are needed to probe into the psychology and contextual factors of GBV and reproductive health in greater detail to provide a more comprehensive explanation of the mechanisms at play. Research is also needed to test the practicality and effectiveness of interventions that aim to prevent and treat GBV as a means for improving reproductive health outcomes in a Vietnamese context.
